# Retinal artery occlusion is associated with compositional and functional shifts in the gut microbiome and altered trimethylamine-N-oxide levels

**DOI:** 10.1038/s41598-019-51698-5

**Published:** 2019-10-25

**Authors:** Denise C. Zysset-Burri, Irene Keller, Lieselotte E. Berger, Peter J. Neyer, Christian Steuer, Sebastian Wolf, Martin S. Zinkernagel

**Affiliations:** 1 Department of Ophthalmology, Inselspital, Freiburgstrasse, CH-3010 Bern, Switzerland; 2 Department of Clinical Research, University of Bern, Murtenstrasse 31, CH-3010 Bern, Switzerland; 3 Swiss Institute of Bioinformatics, Baltzerstrasse 6, CH-3012 Bern, Switzerland; 4 Institute of Laboratory Medicine, Kantonsspital Aarau, Tellstrasse 25, CH-5001 Aarau, Switzerland; 5 Graduate School for Cellular & Biomedical Sciences, University of Bern, Freiestrasse 1, CH-3012 Bern, Switzerland; 6 Department of Chemistry and Applied Biosciences, Institute of Pharmaceutical Sciences, Swiss Federal Institute of Technology (ETH), Vladimir-Prelog-Weg 4, CH-8093 Zürich, Switzerland

**Keywords:** Retinal diseases, Risk factors

## Abstract

Retinal artery occlusion (RAO) is a sight threatening complication of cardiovascular disease and commonly occurs due to underlying atherosclerosis. As cardiovascular disease and atherosclerosis in particular has been associated with compositional alterations in the gut microbiome, we investigated this association in patients with clinically confirmed non-arteritic RAO compared to age- and sex-matched controls. On the phylum level, the relative abundance of *Bacteroidetes* was decreased in patients with RAO compared to controls, whereas the opposite applied for the phylum of *Proteobacteria*. Several genera and species such as *Actinobacter*, *Bifidobacterium spp*., *Bacteroides stercoris*, *Faecalibacterium prausnitzii* were relatively enriched in patients with RAO, whereas others such as *Odoribacter*, *Parasutterella* or *Lachnospiraceae* were significantly lower. Patient’s gut microbiomes were enriched in genes of the cholesterol metabolism pathway. The gut derived, pro-atherogenic metabolite trimethylamine-N-oxide (TMAO) was significantly higher in patients with RAO compared to controls (p = 0.023) and a negative correlation between relative abundances of genera *Parasutterella* and *Lachnospiraceae* and TMAO levels and a positive correlation between relative abundance of genus *Akkermansia* and TMAO levels was found in study subjects. Our findings proposes that RAO is associated with alterations in the gut microbiome and with elevated TMAO levels, suggesting that RAO could be targeted by microbiome-altering interventions.

## Introduction

**Figure 1:**
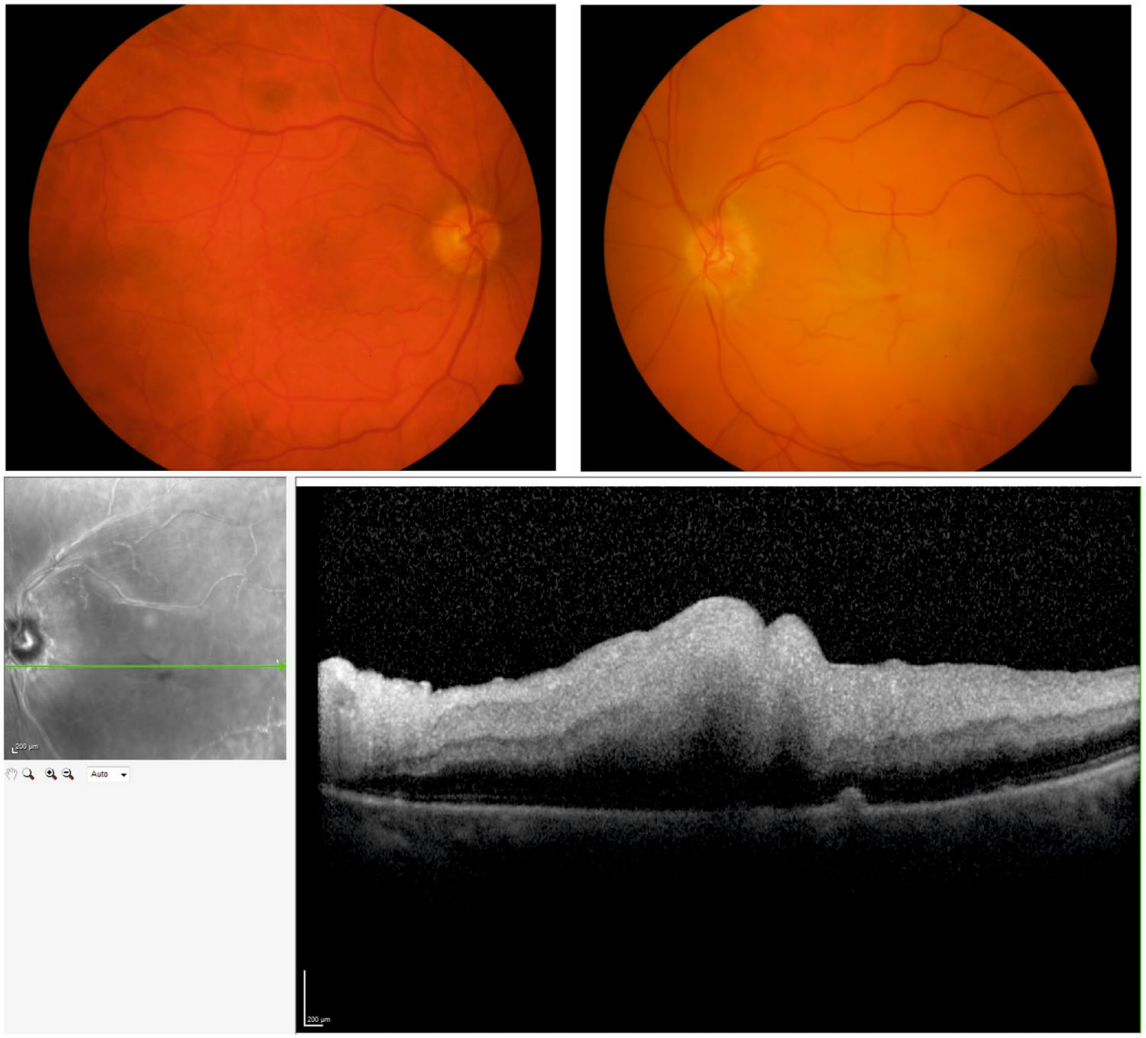
Representative images of a patient with central retinal artery occlusion. Top: Representative color photographs of the unaffected right eye and the left eye with typical features of a central artery occlusion with ischemia and edema of the retina and a cherry red spot. Bottom: Optical coherence tomography (OCT) images of the same patient showing a scan through the right retina. Swelling of the inner retinal layers is evident due to occlusion of the central retinal artery.

Acute retinal arterial occlusive disorders comprise one of the major causes of acute visual loss. Acute occlusion of central or branch retinal arteries termed retinal artery occlusion (RAO), may result in ischemia of the inner retina with initial swelling and eventually atrophy and painless monocular loss of vision (Fig. 1)^1^. The pathogenesis of RAO is primarily thromboembolic, often from carotid plaques, and therefore closely associated with atherosclerosis^2,3^. Factors contributing to atherosclerosis and cardiovascular disease in general arise mainly from environmental sources or a combination of genetic and environmental causes. Diet has been identified as the major risk factor for cardiovascular disease with a positive correlation with total and low-density lipoprotein (LDL) cholesterol and an inverse correlation with high-density lipoprotein (HDL) cholesterol^4,5^. Additional risk factors include elevated blood pressure, cigarette smoking and diabetes mellitus.

Digestion of macronutrients, consisting of carbohydrates, proteins, fats and fibers, results in a myriad of metabolites that enter the circulation. The gut microbiome has been shown to be integral to digestion and contributes to a substantial portion of the variation in blood lipids^6^. Furthermore, it can generate nutrients from substrates that are otherwise not degradable by the host^7^. Alterations in the gut microbiome have been associated with cardiovascular disease and atherosclerosis^8–10^. Furthermore, links between the gut microbiome and diabetes mellitus and obesity have been revealed in the last decade, both of which are associated with atherosclerosis^11–13^. In addition, in retinal diseases such as age related macular degeneration, which is the leading cause of irreversible severe visual loss in the western world and has been associated with atherosclerosis^14^, alterations in the gut microbiota have been described^15,16^. However, a possible link between alterations in the gut microbiota and RAO has so far not been investigated.

The exact mechanisms linking the gut microbiota with the development of atherosclerosis have not yet materialized. Several studies have reported a link between choline diet–induced trimethylamine N-oxide (TMAO) production in the gut and cardiovascular disease^9,10,17^. Dysbiosis in the gut has been shown to lead to increased permeability of the intestine, which in turn causes increased systemic levels of bacterial products resulting in low-grade chronic inflammation^18^. This may directly affect atherogenesis and may lead to the development of insulin resistance with concomitant effects on plasma lipids^19^.

In this study, we performed a systematic analysis of the gut microbiome and associated metabolic pathways and analyzed TMAO levels as well as genes of the TMAO synthesis pathway in patients with symptomatic RAO and controls.

## Results

### Taxonomic characterization of the gut microbiome

**Figure 2:**
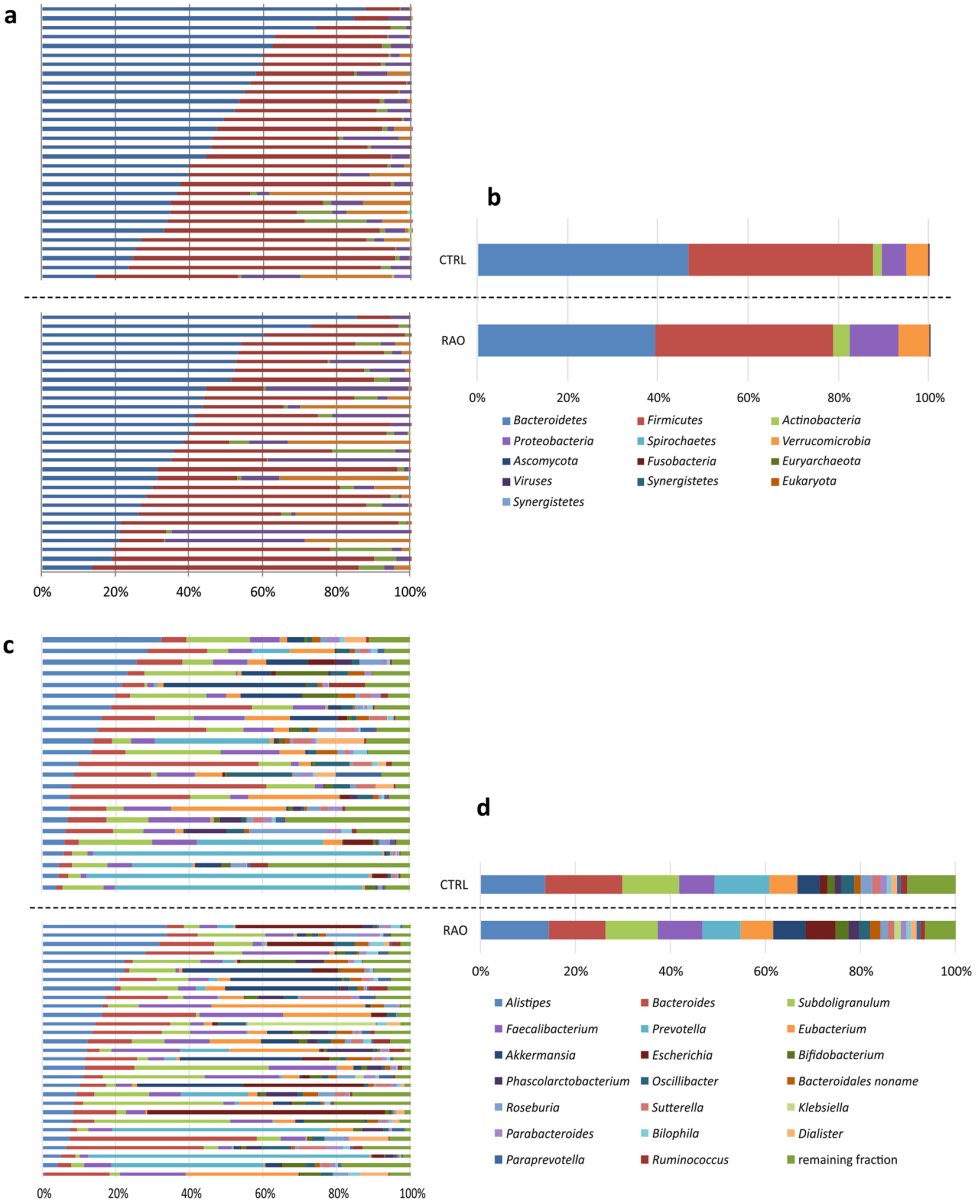
Taxonomic characterization of the gut microbiome. Relative abundances of microbiota at phyla level in all study subjects (**a**) and averaged for study groups (**b**). There is a decrease in relative abundance of *Bacteroidetes* with respect to *Proteobacteria* in the RAO cohort. Relative abundances of microbiota at genus level in all study subjects (**c**) and averaged for study goups (**d**). There is a decrease of *Bacteroides* in the RAO group compared to controls. CTRL, c ontrol (n = 30); RAO, retinal artery occlusion (n = 29).

To investigate if the intestinal microbiome is associated with the occurrence of RAO, we sequenced the gut metagenomes of 29 patients with clinically confirmed non-arteritic RAO and 30 healthy age- and sex-matched controls (Table 1). In total, we generated 1.77 billion 151 bp paired-end reads, i.e. on average, 31 ± 12.6 (s.d.) million reads per sample. After trimming and filtering, we obtained 28 ± 11.7 (s.d.) million non-human high-quality reads per sample for further processing. The majority of the mapped reads were bacterial (99.9 ± 0.10% in patients and 99.7 ± 0.9% (s.d.) in controls) and dominated by the phyla *Bacteroidetes* and *Firmicutes*, followed by *Proteobacteria* and *Actinobacteria* (Fig. 2a,b). The most abundant classes in our cohort were *Bacteroidia* and *Clostridia* consistent with previous observations^20,21^. The microbiome composition was dominated by the genera *Bacteroides* and *Alistipes*, followed by *Subdoligranulum*, *Prevotella* and *Faecalibacterium* (Fig. 2c,d). The most abundant species was *Subdoligranulum species*, followed by *Faecalibacterium prausnitzii*, *Prevotella copri*, *Alistipes putredinis* and *Akkermansia muciniphila*.

**Table 1:** Characteristics of study patients.

Feature	Patients (n = 29)	Controls (n = 30)	P value RAO vs CTRL
Males (n)	15	14	0.80^∆^
Age (years)	69.4 ± 1.9	69.0 ± 1.7	0.93*
Current smoker (n)	5	3	0.47^∆^
Previous smoker (n)	12	10	0.60^∆^
BMI (kg/m^2^)	27.6 ± 0.8	25.8 ± 0.7	0.10*

BMI, body mass index; CTRL, control; RAO, retinal artery occlusion. Data are mean ± s.d., ^∆^Fisher’s exact test, *Welch’s t test.

### Enterotypes and distinct microbial composition in the cohort

**Figure 3:**
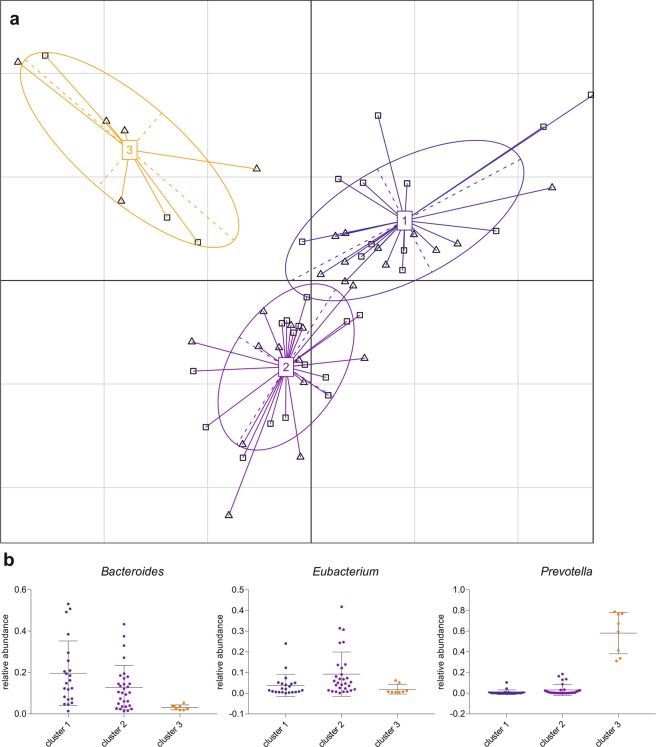
Gut enterotypes in the cohort. Three enterotypes exist in the cohort based on the abundance of microbial genera. Patients (n = 29) and controls (n = 30) are denoted by squares (□) and triangles (∆), respectively (**a**). Boxplots represent the abundance of *Bacteroides*, *Eubacterium* and *Prevotella*, the proposed drivers of the three enterotypes (**b**). Blue is enterotype 1, purple is enterotype 2 and orange is enterotype 3.

A previous study has suggested that the human intestinal microbiome can be divided into three enterotypes of distinct microbial composition^20^. According to this study, we calculated the Jensen-Shannon distance of the genus abundances and clustered the samples with partitioning around medoids (PAM). Graphical interpretation in Fig. 3a was done using between class analysis (BCA) of the genus compositions as suggested in Arumugam *et al*.^20^, which visualizes results from principal component analysis (PCA) and clustering. The enterotypes were characterized by the following contributors at genus level: *Bacteroides* was the driving genus of enterotype 1, *Eubacterium* of enterotype 2 and *Prevotella* contributed to enterotype 3 (Fig. 3b). Applying Fisher’s exact test, no association between the enterotypes and the disease status could be detected, showing an uniform distribution of the samples across the three enterotypes.

**Figure 4:**
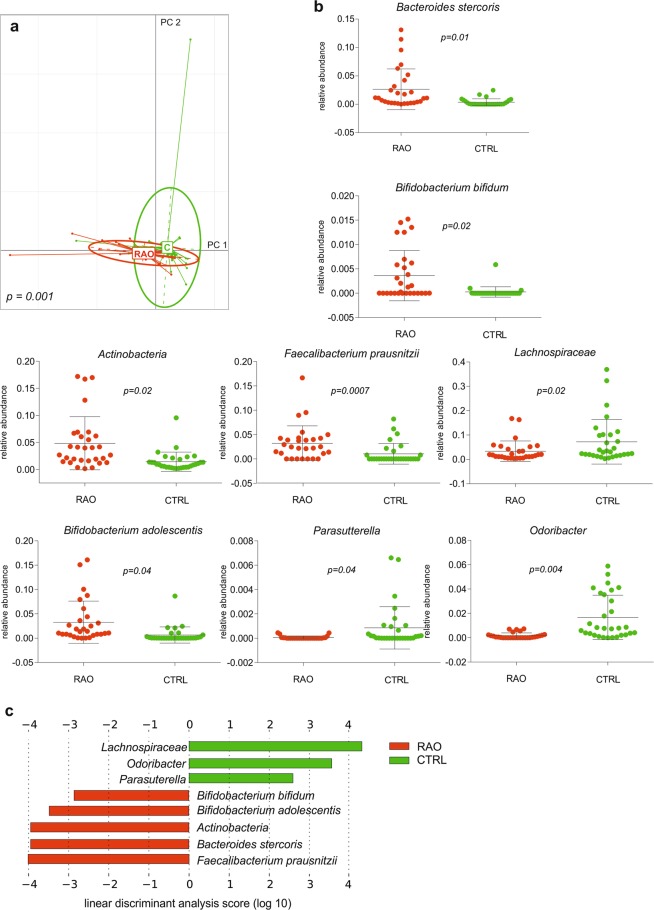
Distinct microbial composition between retinal artery occlusion (RAO) patients and control. (**a**) Principal component analysis of microbial species abundance grouped patients and controls separately, with PERMANOVA confirming a significant difference between the groups (p = 0.001). (**b**) Box plots representing the mean abundance +/− s.d. of bacterial genera and species associated with RAO (Kruskal-Wallis test, p < 0.05). (**c**) LDA (linear discriminant analysis) score plot of differentially abundant taxonomic features among groups (LDA score for discriminative features >2.0). Red is patients (RAO, n = 29), green is controls (CTRL, n = 30).

A PCA with the health status as grouping variable showed that differences in microbial species abundance separated the patient group from the control group, with permutation multivariate analysis of variance (PERMANOVA) confirming a significant p value of 0.001 (Fig. 4a). To identify taxa that are different in relative abundance between RAO and controls, the linear discriminant analysis effect size algorithm LEfSe was applied on the previously identified microbiome. The class *Actinobacteria* and the species *Bifidobacterium adolescentis*, *Bifidobacterium bifidum*, *Bacteroides stercoris* and *Faecalibacterium prasnitzii* were enriched in RAO, whereas the family *Lachnospiraceae* and the genera *Odoribacter* and *Parasutterella* were enriched in controls (p < 0.05, Kruskal-Wallis test; Fig. 4b,c).

### Metabolic features of altered gut microbiome

**Figure 5:**
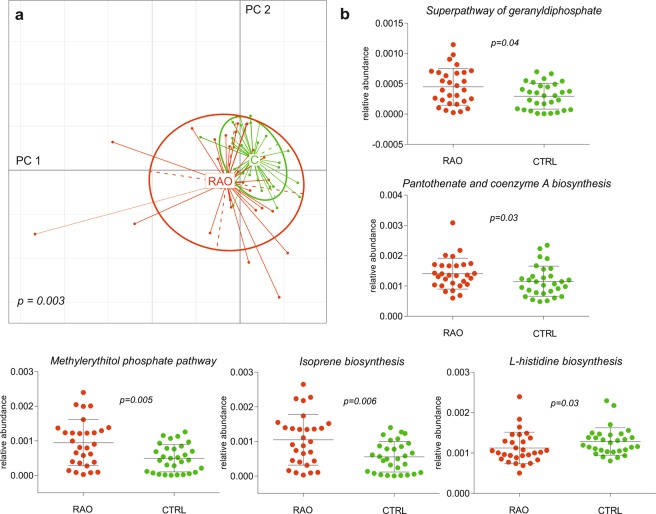
Species-specific microbial pathways associated with retinal artery occlusion (RAO). (**a**) Principal component analysis of microbial pathway abundance grouped patients and controls separately, with PERMANOVA confirming a significant difference between the groups (p = 0.003). (**b**) Box plots representing the abundance of pathways associated with RAO and illustration of the respective pathways which differed in patients with RAO and control subjects (Kruskal-Wallis test, p < 0.05). Red is patients (RAO, n = 29), green is controls (CTRL, n = 30).

To describe the metabolic functions of the microbiome, the Metagenomic Phylogenetic Analysis tool HUMAnN2 was applied on each sample separately based on the identified taxonomic profiles. Resulting organism-specific gene hits were functionally assigned to pathways using MinPath and their relative abundances were evaluated. A PCA with the health status as grouping variable showed that differences in abundance of metabolic functions separated the patient group from the control group, with PERMANOVA confirming a significant p value of 0.003 (Fig. 5a). In total 5 abundant metabolic pathways and 10 gene families (i.e. occurring in at least 30 samples) were identified differing between RAO patients and controls (p < 0.05, Kruskal-Wallis test), illustrating that there were functional aspects of the gut microbiome associated with RAO. Enriched metabolic functions in the microbiome of patients and controls were assessed by integrating the gene families with metabolic pathways. We used UniProt for protein identification, and based on the KEGG PATHWAY Database we identified pathways involved in secondary metabolite biosynthesis. The pantothenate and coenzyme A biosynthesis (map 00770) was the highest abundant metabolic pathway and was enriched in the gut microbiome of RAO patients. Coenzyme A is an essential cofactor for cell growth and is involved in many metabolic reactions including the synthesis and degradation of fatty acids^23^. Consequently, we also found several metabolites enriched in the microbiome of patients that were involved in metabolic reactions such as isoprene biosynthesis, pathway of geranylgernaly-diphosphate and methylerythritol phosphate, whereas metabolites involved in the L-histidine biosynthesis pathway were enriched in the microbiome of controls (Fig. 5b).

### Concentrations of trimethylamine-N-oxide (TMAO)

**Figure 6:**
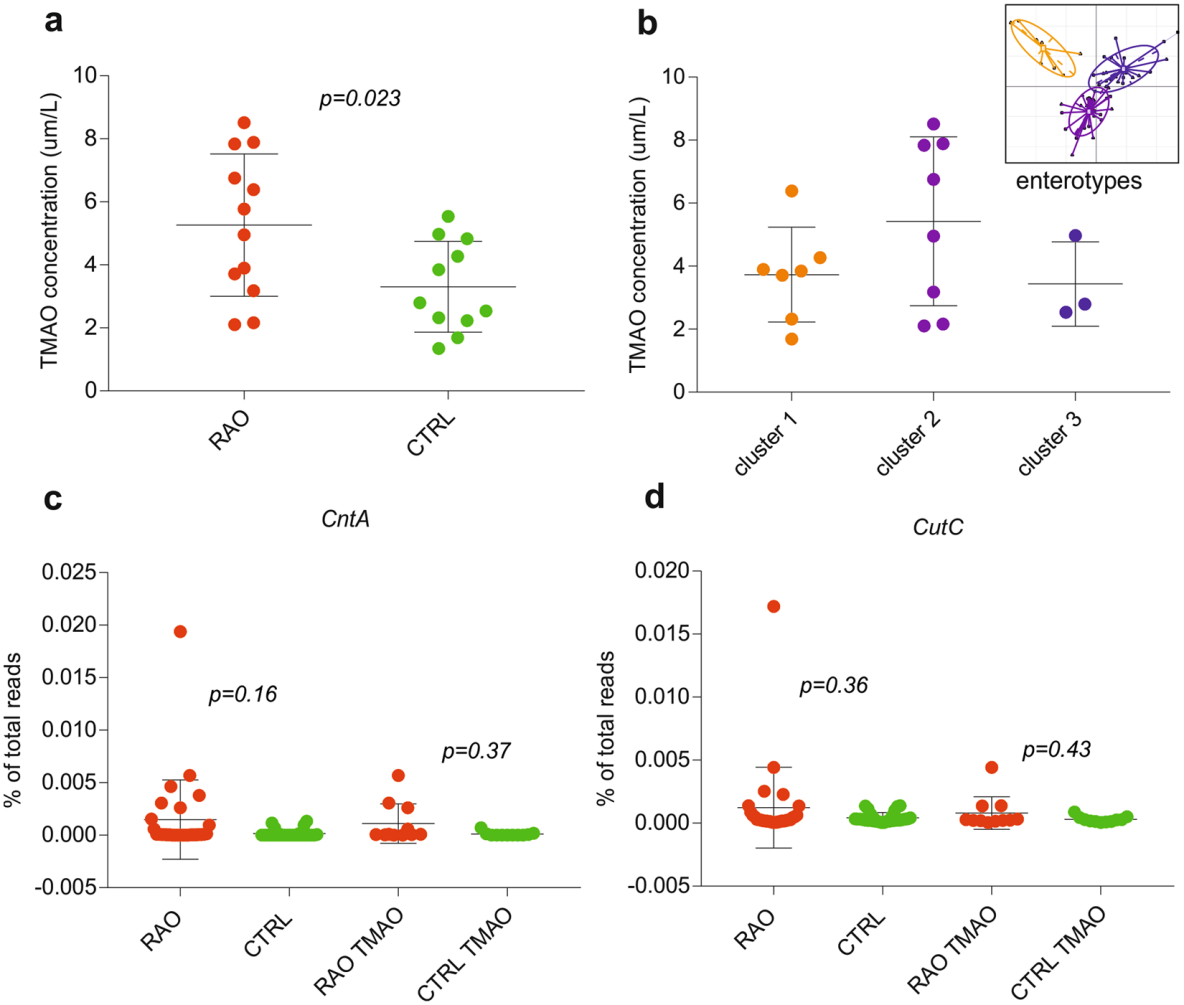
Trimethylamine-N-oxide (TMAO) concentration associated with retinal artery occlusion (RAO). (**a**) The TMAO concentration was significantly higher in a subgroup of 12 RAO patients compared to 11 controls (Fisher’s exact test, p = 0.023). (**b**) No significant difference in the TMAO concentration among the three enterotypes has been found (Kruskal Wallis test, p > 0.05). Abundance of carnitine oxygenase (*cntA*, **c**) and choline trimethylamine-lyase (*cutC*, **d**) in patients (RAO, n = 29) and controls (CTRL, n = 30) as well as in the TMAO subgroup of 12 patients (RAO TMAO) and 11 controls (CTRL TMAO) as percentage of total reads. Obtained p values (Fisher’s exact test) are indicated. Red is patient (RAO), green is controls (CTRL), blue is enterotype 1, purple is enterotype 2 and orange is enterotype 3.

To assess the TMAO concentration and correlation to metagenomic data, a subgroup analysis with 12 RAO patients and 11 age- and sex-matched controls was performed. The TMAO concentration was significantly higher in patients with RAO compared to controls (5.26 ± 2.26 µmol/L versus 3.31 ± 1.44 µmol/L (s.d.), p = 0.023; Fig. 6A). Multivariate analysis using MaAsLin found a negative correlation between relative abundances of genera *Parasutterella* and *Lachnospiraceae* and TMAO levels and a positive correlation between relative abundance of genus *Akkermansia* and TMAO levels in study subjects. However, no significant differences in the TMAO level among the three enterotypes of Fig. 3 could be found (p > 0.05, Kruskal-Wallis test, Fig. 6B). In order to examine the TMAO-forming potential of microbes in the cohort as well as in the TMAO subgroup, metagenomic reads were BLASTed against a database for key genes of the TMAO synthesis pathway, encoding choline trimethylamine-lyase (*cutC*) and carnitine oxygenase (*cntA*)^24^. The abundances of key genes of both pathways were elevated in the patient group (mean values of 0.00097% and 0.0011% for *cutC* and *cntA*, respectively) compared to controls (mean values of 0.00043% and 0.00017% for *cutC* and *cntA*, respectively) as percentage of total reads. However, these observed differences were statistically not significant (p > 0.05, Fisher’s exact test, Fig. 6c,d).

## Discussion

In this study, we identified several compositional and functional alterations of the gut microbiome that may be related to RAO which is closely associated with atherosclerosis. Atherosclerotic disease is characterized by lipid accumulation and recruitment of macrophages to the arterial wall forming plaques. It has been shown that atherosclerotic plaques contain bacterial DNA with phylotypes common to the gut microbiota and that the amount of bacterial DNA in the plaque correlate with inflammation^25^. Furthermore, Karlsson *et al*. suggested that the gut microbiome is associated with the inflammatory status of the host and patients with symptomatic atherosclerosis exhibit characteristic changes in the gut microbiota, including a higher abundance of the genus *Collinsella* in patients and of *Roseburia* and *Eubacterium* in healthy controls^8^.

The healthy human gut microbiome is known to be stable over time^26,27^. Generally, the phyla *Bacteroidetes* and *Firmicutes* dominate the gut microbial community followed by *Proteobacteria* and *Actinobacteria*^28^ as observed in our cohort. However, diseases associated with metabolism and immune responses result in an imbalanced unstable state of the microbiome, called dysbiosis. Dysbiosis is well documented in metabolic disorders including an increase in relative abundance of *Firmicutes* with respect to *Bacteroidetes*^29,30^. Furthermore, an elevated prevalence of *Proteobacteria* has been proposed as a diagnostic marker for dysbiosis and risk of disease^28^. In our study, there is a decrease of *Bacteroidetes* (39.2% in patients vs 46.7% in controls) with respect to *Proteobacteria* (10.8% in patients vs 5.4% in controls) in RAO patients (Fig. 2b), suggesting a correlation between atherosclerotic disease and dysbiosis.

At the taxonomical level, we observed associations between RAO and microbiome composition, and furthermore, PCA and PERMANOVA on microbial species abundance confirmed a separation of patients and controls by microbiome composition (Fig. 4a). Previous studies have identified a higher abundance of *Collinsella*, a genus of *Actinobacteria* in the gut of patients with symptomatic atherosclerosis, which is in keeping with our data. Several studies have identified bacterial DNA in human atherosclerotic plaque samples of which a high proportion could be attributed to *Actinobacteria*^25,31^. DNA contained in atherosclerotic plaques have been shown to resemble the phylotypes common to the gut microbiota and therefore support the hypothesis that the gut microbiota can be sources for atherosclerotic plaque-associated bacteria^25^.

The higher abundance of two *Bifidobacterium species* in our RAO cohort is surprising. *Bifidobacterium species* have received considerable attention mainly as common probiotics and have been implicated to have a beneficial effect on blood lipid concentrations. A meta-analysis of 30 randomized controlled trials revealed that probiotic bacteria supplementation resulted in significantly lower concentrations of total cholesterol and LDL cholesterol. However, probiotic supplementation did not change levels of HDL cholesterol or triglycerides, both of which are associated with the development of atherosclerosis^32^. On the other hand, higher proportions of *Bifidobacterium species* have been implicated in inflammatory bowel disease which in turn is associated with early atherosclerosis^33–35^.

However, sequencing the microbial genes is not enough because the presence of DNA alone does not necessarily translate into protein synthesis and function affecting the host. Functional annotation analysis indicated that metabolic pathways are enriched or decreased in patients with RAO. Furthermore, PCA and PERMANOVA on abundance of metabolic functions confirmed a separation of patients and controls by functional features of the gut microbiome (Fig. 5a). The isoprene biosynthesis pathway, also known as the mevalonate pathway, was enriched in patients with RAO. This pathway converts mevalonate into sterol isoprenoids, such as cholesterol, and is best known as the target of statins, a class of cholesterol lowering drugs. Likewise, the methylerythritol phosphate pathway was enriched in RAO patients. This pathway is a source of isoprene units in most bacteria that are key intermediates in the pathway from acetate to cholesterol^36^.

In our study, we found a significant difference in TMAO levels between patients with RAO and controls. TMAO is believed to be pro-atherogenic and has been associated with cardiovascular risks by promoting foam cell formation and interfering with cholesterol transport^37,38^. In humans, TMAO is produced from choline and carnitine by the gut microbiota. The main producers of TMAO derive from the two phyla *Firmicutes* and *Proteobacteria* and include *Escherichia fergusonii*, *Proteus penneri*, *Providencia rettgeri*, *Anaerococcus hydrogenalis*, *Clostridium asparagiforme*, and *Edwardsiella tarda*^39,40^. In our study, we found a negative association between the TMAO concentration and *Parasutterella* and *Lachnospiraceae*, which is consistent with the upregulation of these genera in controls (Fig. 4). Moreover, the positive correlation found between the TMAO concentration and *Akkermansia* is consistent with previous observations^39^. However, whether increased levels of TMAO are quintessentially causative for atherosclerosis or whether they merely represent a biomarker of differences in the gut microbiome remains to be investigated.

Our findings indicate that patients with RAO may have alterations in gene expression pathways related to cholesterol production. This is especially interesting in view of recent findings that enteric microbiome metabolites have been shown to correlate with response to simvastatin treatment^41^. Statins are HMG-CoA reductase inhibitors and are commonly used to reduce plasma levels of LDL cholesterol in order to prevent coronary artery disease^42^. Further studies are needed to confirm functional differences in the gut microbiome by correlating metabolic profiles and quantification of messenger RNA in patients with atherosclerosis. However, as the prognostic information is limited because of possible confounders due to genetic background and nutritional and environmental habits of patients and controls, further studies are necessary. On its own, our findings are hypothesis generating for further trials investigating the impact of the gut microbiome on atherosclerosis mediated ophthalmic diseases.

## Conclusions

This study shows compositional and functional alterations in the gut microbiome and evelated TMAO levels in patients with RAO, and therefore may have implications on preventative strategies for this vision threatening disease.

## Methods

### Study design and recruitment

Participants (n = 59) were recruited from the Department of Ophthalmology of the University Hospital Bern (Inselspital), Switzerland. The study was conducted in accordance with the Declaration of Helsinki and approved by the Ethics Committee of the Canton of Bern (ClinicalTrials.gov: NCT02438111). After receiving oral and written information, all participants gave written informed consent to participate in the study. All participants were subjected to an ophthalmic examination including optical coherence tomography and standard fundus color photography. Patients (n = 29) had clinically confirmed non-arteritic RAO and the control group (n = 30) was selected to represent an age- and sex-matched group with no sign of RAO. We tested for differences between the two groups in a range of demographic values using either Welch’s t test (for age and BMI) or Fisher’s exact test (for gender and smoking; Table 1). Exclusion criteria for both groups were chronic inflammatory and gastrointestinal diseases (including previous surgery in the gastrointestinal tract) and use of systemic antibiotics within the last three months.

### Sequencing and data control

Stool samples were brought chilled to the study center within 16 hours after fecal output where they were immediately frozen at −20 °C. Metagenomic DNA was isolated from up to 200 mg of stool sample using the PSP^®^ Spin Stool DNA *Plus* kit (Stratec Biomedical AG, Beringen, Switzerland) according to the manufacturer’s protocol with an integrated RNA digestion step using 100 mg/ml RNase A (Qiagen, Hombrechtikon, Switzerland). The DNA was brought to the Next Generation Sequencing Platform of the University of Bern, Switzerland, for metagenomic shotgun sequencing. The TruSeq DNA PCR-Free Library Preparation kit was used for library preparation for sequencing following standard pipelines of the Illumina HiSeq 3000 platform with up to ten samples pooled in one lane. The resulting 150 bp paired-end reads were quality filtered with Trimmomatic v.0.32^43^. To remove sequences of human origin, all reads were mapped to the human reference genome hg19 using Bowtie2 v.2.2.4^44^ and only the unmapped reads were used for further analysis.

### Microbial and functional profiling

For taxonomical analysis, the high-quality non-human reads were mapped against a set of clade-specific marker sequences using the Metagenomic Phylogenetic Analysis tool v.2.6.0 (MetaPhlAn2) and the marker database v.20^45^ using default settings. In order to provide the relative abundance of each taxonomic unit, Bowtie2 v.2.2.4 was applied for alignment followed by normalization of the total number of reads in each clade by the nucleotide length of its marker.

To describe the metabolic potential of the identified microbes, the HMP (Human Microbiome Project^46^) Unified Metabolic Analysis Network (HUMAnN2 v.0.11.0^22^) was applied using default settings. HUMAnN2 assesses the abundance (reads per kilobase; RPK) of gene families and pathways in each sample, to provide a functional interpretation of the metagenomic sequences. HUMAnN2 was run for each sample separately, including information of the taxonomic profiles from MetaPhlAn2. For nucleotide-level searches, Bowtie2 v.2.2.4 was applied to map reads to the functionally annotated pangenome database ChocoPhlAn. All unmapped reads were used for translated searches against the universal protein reference database UniRef90^47^ applying Diamond v.0.8.37^48^. The resulting organism-specific gene hits were assigned to pathways using MinPath v.1.2^49^, finally providing a set of pathways including their abundances. The R package ade4^50^ was used to perform PCA providing global analysis of microbial species abundance and pathway abundance between RAO and controls. A visualization of the individual samples grouped by case and control is provided in Figs 4a and 5a. Permutation multivariate analysis of variance (PERMANOVA) using the R package vegan^51^ was assessed with 1000 permutations to calculate a p value for separation.

To identify taxonomic and functional features with significantly different relative abundances in RAO and controls, the linear discriminant analysis effect size (LEfSe) algorithm^52^ was applied using default settings. A difference was considered to be statistically significant if p < 0.05 (KruskalKruskal-Wallis test) and LDA score ≥2.

### Measurement of trimethylamine-N-oxide (TMAO)

TMAO was obtained from Alfa Aesar (Kandel, Germany), ammonium formate and formic acid from Sigma (Buchs, Switzerland) and all reagents were of the highest analytical grad available. Solvents (water, methanol, acetonitrile) were also obtained from Sigma and of mass spectrometry grade. Blood plasma (EDTA) samples were obtained from a fasting subgroup including 12 RAO patients and 11 controls and stored at −80 °C until analysis which was performed as published previously^53^. In short: Samples were thawed at room temperature, 25 µL aliquots were transferred to reaction cups and diluted with 225 µL of methanol containing the deuterated internal standard (TMAO-d9, Cambridge Isotope Laboratories, Andover, USA). After ten minutes and complete protein precipitation, the cups were briefly agitated and spun in a micro centrifuge at 15 000 *g* for 10 minutes. Chromatographic analysis was done using an UltiMate 3000 chromatographic system (Thermo Fisher Scientific, Reinach, Switzerland) coupled to a triple quadrupole mass spectrometer (Sciex QTRAP 5500, Darmstadt, Germany). Supernatant was injected onto a hydrophilic interaction column (Phenomenex, Luna 3 µm HILIC 200 Å, 150 × 4.6 mm). Separation was done using a linear gradient starting at 1.5 minutes post-injection from 100% of mobile phase A (10 mM ammonium formate in acetonitrile) to 90% of mobile phase B (10 mM ammonium formate in water, adjusted to pH 4 with formic acid) within seven minutes at a flow rate of 750 µL/min. This ratio was kept constant for another two min and the system was re-equilibrated until a total run time of 12.5 min. Analytes were detected by monitoring specific mass transitions (MRM mode). Concentrations were reported in µmol/L and compared between patients and controls using Fisher’s exact test. To assess the association between microbial abundance and TMAO concentration, multivariate association with linear models (MaAsLin) for multivariate analysis^54^ applying an additive general model was used. Significant association was considered below a q value threshold of 0.20 after adjusting for false discovery rate (FDR). To identify genes of the TMAO synthesis pathway in our metagenomics data, reads were mapped against the *cntA/cutC* database provided by Rath *et al*.^24^ using the BWA-MEM algorithm of the Burrows-Wheeler Alignment tool v.0.7.17. The number of mapped reads was counted by the Samtool flagstat v.1.8.

## Data Availability

The datasets supporting the conclusions of this article are available in the European Nucleotide Archive under accession number PRJEB24557.
